# Increased risk of lymphoid malignancy in patients with herpes zoster: a longitudinal follow-up study using a national cohort

**DOI:** 10.1186/s12885-019-6349-y

**Published:** 2019-11-27

**Authors:** Hyo Geun Choi, James L. Zehnder, Young Kyung Lee, Hyun Lim, Miyoung Kim

**Affiliations:** 1 Department of Otorhinolaryngology-Head & Neck Surgery, Hallym University College of Medicine, Anyang, Republic of Korea; 2 Department of Pathology, Stanford University, Stanford, CA USA; 3 Division of Hematology, School of Medicine, Stanford University, Stanford, CA USA; 4 Department of Laboratory Medicine, Hallym University College of Medicine, Anyang, Republic of Korea; 5 Department of Internal Medicine, Hallym University College of Medicine, Anyang, Republic of Korea; 6 Department of Laboratory Medicine, Hallym University Sacred Heart Hospital, 22, Gwanpyeong-ro 170 beon-gil, Dongan-gu, Anyang-si, Gyeonggi-do 14068 South Korea

**Keywords:** Lymphoma, Herpes zoster, Risk, Korea

## Abstract

**Background:**

The association between herpes zoster and the risk of lymphoid neoplasms in Asian populations has not yet been established. We performed a longitudinal follow-up study using a nationwide cohort to assess the risk of lymphoid neoplasms arising after herpes zoster infection in the adult Korean population.

**Methods:**

Data from participants ≥20 years of age who were registered in the Korean National Health Insurance Service-National Sample Cohort database between 2002 and 2013 were collected. We extracted the data of participants with herpes zoster (*n* = 59,495) as well as those of matched references at a ratio of 1:4 (*n* = 237,980) and investigated the subsequent occurrence of lymphoid neoplasms. A stratified Cox proportional hazards model was used to calculate unadjusted hazard ratios (HRs) as well as those adjusted for the Charlson comorbidity index score.

**Results:**

The rate of lymphoid neoplasms was higher in the herpes zoster group (0.15% [90/59,495]) than in the reference group (0.08% [212/237,980], *P* < 0.001). The unadjusted and adjusted HRs of herpes zoster in patients with lymphoid neoplasms were 1.68 (95% confidence interval [CI] = 1.31–2.15) and 1.58 (95% CI = 1.23–2.02), respectively (*P* < 0.001 for both). On subgroup analyses according to age and sex, herpes zoster was associated with an increased risk of lymphoid neoplasms in all subgroups; the adjusted HRs were 1.53 (95% CI = 1.05–2.24) for patients < 60 years old, 1.58 (95% CI = 1.14–2.20) for patients ≥60 years old, 1.64 (95% CI = 1.16–2.31) for men, and 1.51 (95% CI = 1.06–2.16) for women (*P* < 0.05 for all). On subgroup analysis of lymphoid neoplasm subtypes, herpes zoster was associated with the risk of Hodgkin’s disease (adjusted HR: 3.23 [95% CI = 1.17–8.93]) and multiple myeloma/malignant plasma cell neoplasms (adjusted HR: 2.17 [95% CI = 1.33–3.54]) (*P* < 0.05 for both).

**Conclusion:**

Herpes zoster is associated with lymphoid neoplasm development in the Korean population irrespective of age and sex. The risks of Hodgkin’s disease and plasma cell neoplasms are significantly elevated in patients with herpes zoster.

## Background

Herpes zoster is a viral disease characterized by painful and pruritic vesicles in a dermatomal distribution [1, 2]. It is caused by the reactivation of latent varicella zoster virus infection, which is associated with a decline in cell-mediated immunity [3–5]. Previous studies have suggested an association between herpes zoster and lymphoid neoplasms because both conditions are associated with impaired immunity [6–13]. Moreover, it was posited that herpes zoster could serve as an indicator of occult disease in patients with different types of cancers, including those of the gastrointestinal tract, lung, bone, and soft tissues, as well as hematologic malignancies [6–9]. The importance of herpes zoster has particularly been recognized in lymphoid neoplasms given that the causal relationships between such neoplasms and viral infections have already been established [10–13]. The Epstein-Barr virus is associated with Hodgkin’s lymphoma, Burkitt lymphoma, extranodal natural killer/T-cell non-Hodgkin’s lymphoma (NHL), and posttransplant lymphoproliferative disorder [10]. Moreover, human T-cell leukemia/lymphotropic virus type 1 is a causative agent of adult T-cell leukemia/lymphoma [10], while the human immunodeficiency virus is associated with acquired immunodeficiency syndrome-associated NHLs [10]. Hepatitis B and C viruses have also been associated with different types of lymphoid neoplasms [11–13]. As such, clarifying the association between herpes zoster and subsequent lymphoid neoplasms would be helpful in elucidating the pathogenesis of lymphoid neoplasms and facilitating their surveillance and early diagnosis.

Most studies of the association between herpes zoster infection and lymphoid neoplasms investigated whether the former occurs in patients with the latter, while studies that explored the risk of lymphoid neoplasm occurrence in patients already infected with herpes zoster are relatively scarce and have not produced definitive conclusions [1, 14]. Moreover, most case-control studies are from Western countries; hence, their findings may not necessarily be applicable to populations of other countries owing to ethnic and geographic differences. Indeed, it has been reported that the incidences of herpes zoster and lymphoid neoplasms differ between Asian and Western populations, as do the relative frequencies of the various subtypes of lymphoid neoplasms. The incidence of herpes zoster is reported to be 10.4 per 1000 person-years in Korea [15], whereas that in Western countries was determined to be 4–4.5 per 1000 person-years [16]. The incidence rates of follicular lymphoma, chronic lymphocytic leukemia, and Hodgkin’s disease were lower [17], but the incidence rates of marginal zone lymphoma and “extranodal natural killer/T-cell lymphoma, nasal type” were higher in Asian populations than in Western populations [18–20]. Incidences in Korea showed trends that were similar to those in the rest of Asia [21].

Despite the epidemiologic importance of understanding the association between herpes zoster and the subsequent risk of lymphoid neoplasms, this association has not been sufficiently explored in Asian populations to date. To that end, we performed a longitudinal follow-up study using a nationwide population-based dataset with a maximum follow-up period of 12 years to explore the association between herpes zoster and the subsequent risk of lymphoid neoplasms in South Korean adults. Our mainstream analysis was of a cohort of participants with herpes zoster and their matched references. To confirm the result of mainstream analysis, we performed 3 additional studies including one with a 6-month wash-out period to verify the chronology of antecedent herpes zoster and subsequent lymphoid neoplasms, a full cohort study with no matching of reference participants to minimize information loss, and a nested case-control study to investigate any previous history of herpes zoster in patients with lymphoid malignancies and their matched references.

## Methods

### Study population and data collection

The ethics committee of Hallym University approved the use of these data (2017-I102). The requirement for written informed consent was waived by the Institutional Review Board.

This national cohort study relied on data from the Korean Health Insurance Review and Assessment Service (HIRA)-National Sample Cohort. The dataset included 1,125,691 participants with 114,369,638 medical claim codes; these data were described in detail in our previous studies [22, 23].

### Participant selection

Among the participants who were diagnosed with herpes zoster (International Statistical Classification of Diseases and Related Health Problems, tenth edition [ICD-10]: B02), only those who were treated for herpes zoster ≥2 times or who were treated with antiviral medication ≥1 time(s) were selected. The participants were followed for up to 12 years.

Participants with lymphoid neoplasms were defined as follows. First, those with the ICD-10 codes C81 (Hodgkin’s disease), C82 (follicular [nodular] non-Hodgkin’s lymphoma), C83 (diffuse non-Hodgkin’s lymphoma); C84 (peripheral and cutaneous T cell lymphomas), C85 (other and unspecified types of non-Hodgkin’s lymphoma), C88 (malignant immunoproliferative diseases), C90 (multiple myeloma and malignant plasma cell neoplasms), and C91 (lymphoid leukemia) were identified. Next, only those who were treated for lymphoid neoplasms ≥3 times or who were treated with chemotherapy or radiation therapy were selected.

**Fig. 1:**
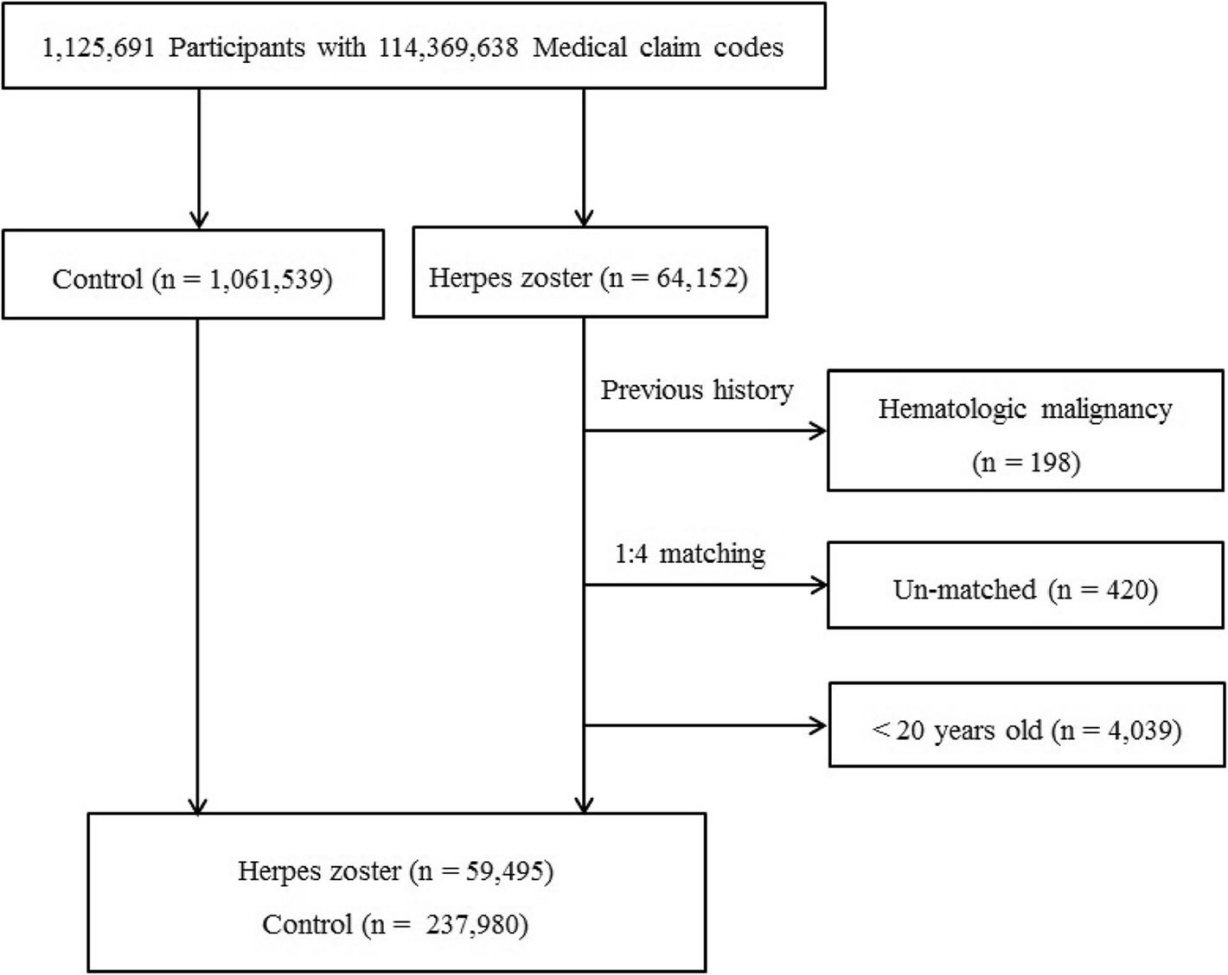
A schematic illustration of the participant selection process used in the present study. Of a total of 1,125,691 participants, 59,495 with herpes zoster and 237,980 controls were matched 1:4 for age, sex, income, and region of residence

Except for analyses that encompassed the full cohort, we used matched reference individuals at a ratio of 1:4. For the mainstream cohort study, participants diagnosed with herpes zoster between 2002 and 2013 were selected first (*n* = 64,152), followed by the matched reference participants who had no record of being diagnosed with herpes zoster during the same period. We set the index date as that of the diagnosis of herpes zoster. The references were selected from the remaining 1,061,539 individuals in the dataset cohort; the matches were screened for age, group, sex, income, and region of residence, and participants in this group were followed beginning from the same index date as that of their matched herpes zoster-infected subjects. The follow-up duration was calculated from the index date to the date of death or study termination (December 31, 2013), and the study subjects were followed until death or censoring. To prevent selection bias, the reference group participants were numbered randomly and were then selected in numerical order. It was assumed that the matched reference participants became involved with the study at the same time as each matched herpes zoster participant (the index date). Therefore, reference participants who died before the index date were excluded. In both the herpes zoster and reference groups, participants with a history of hematologic neoplasms (lymphoid neoplasms and acute leukemia) or of any type of solid cancers before the index date were excluded. As such, 198 participants in the herpes zoster group were excluded, as were 420 for whom we could not find matched controls and 4039 who were under 20 years of age. Ultimately, 1:4 matching produced 59,495 participants with herpes zoster and 237,980 matched references (Fig. 1).

To ensure the actual sequence of herpes zoster and lymphoma diagnosis (i.e., whether herpes zoster truly preceded lymphoid neoplasms among our participants), we additionally investigated the incidences of lymphoid neoplasms at least 6 months after the detection of herpes zoster.

A full-cohort analysis that was not subject to a matching process was also performed to prevent information loss; this analysis included 59,926 participants with herpes zoster and 689,524 reference participants.

Lastly, we applied another approach to compensate for information loss. A nested case control study was performed in which any previous history of herpes zoster was investigated in patients with lymphoid neoplasms and their 1:4 matched reference participants. The matching process was identical to that described above except that participants with lymphoid neoplasms were selected first, and their matched reference participants (i.e., those without lymphoid neoplasms) were selected afterwards (in a 1:4 ratio) using the abovementioned matching criteria (including that for assigning the index date for each participant). Through this process, 1249 participants with lymphoid neoplasms as well as 4996 matched lymphoid neoplasm-free references were selected.

### Variables

Subjects were classified into 14 age groups using 5-year intervals: 20–24, 25–29, 30–34…, and 85+ years old. The income groups were initially divided into 41 classes (1 with health aid, 20 with self-employment-based health insurance, and 20 employment health insurance classes); these groups were recategorized into 5 classes (1 [lowest income] to 5 [highest income]). The regions of residence were divided into 16 areas based on the administrative district and were then regrouped into urban (Seoul, Busan, Daegu, Incheon, Gwangju, Daejeon, and Ulsan) and rural (Gyeonggi, Gangwon, Chungcheongbuk, Chungcheongnam, Jeollabuk, Jeollanam, Gyeongsangbuk, Gyeongsangnam, and Jeju) areas.

The Charlson comorbidity index (CCI) was used for 15 comorbidities as a continuous variable (0 [no comorbidity] through 23 [multiple comorbidities]), except for neoplasms [24].

### Statistical analyses

Chi-square tests were used to compare the general characteristics between 2 groups. Variables compared between the herpes zoster and reference groups included age, sex, income, region of residence, CCI score, and the prevalence of lymphoid neoplasms. Those compared between the lymphoid neoplasm group and its reference group included age, sex, income, region of residence, CCI score, and the prevalence of herpes zoster.

Unadjusted and adjusted hazard ratios (HRs) of herpes zoster infection for lymphoid neoplasms were calculated using a stratified Cox proportional hazards model within a time scale of months. Follow-up analyses (from the index date to that of death, event occurrence [i.e., diagnosis of lymphoid neoplasm], or the follow-up end-date [31 Dec. 2012]) were completed for both the herpes zoster and reference groups; moreover, the proportional hazards assumption between the 2 groups was verified prior to using this model. Unadjusted and adjusted odds ratios (ORs) of lymphoid neoplasms in patients with a previous history of herpes zoster were calculated using a conditional logistic regression analysis; the model was adjusted for the CCI score, and patients were stratified according to age, sex, income, and region of residence. The 95% confidence intervals (CIs) were calculated. For subgroup analyses, we divided the participants by age (< 60 vs. ≥60 years), sex, and lymphoid neoplasm subtypes.

Two-tailed analyses were conducted, and *P*-values less than 0.05 were considered indicative of significance. The statistical analyses of the data were completed using SPSS version 21.0 (IBM Corp., Armonk, NY, USA), and results are presented as the outcomes of chi-square tests and their *P*-values. Moreover, the HRs and ORs (with their 95% CIs and P-values) that were calculated using the stratified Cox proportional hazards model and conditional logistic regression analyses, respectively, are reported.

## Results

The rate of subsequent lymphoid neoplasms was higher in the herpes zoster group (0.15% [90/59,495]) than in the reference group (0.08% [212/237,980], *P* < 0.001, Table 1). The general characteristics of the participants (i.e., age, sex, income group, and region of residence) were exactly the same in terms of the incidence of the diseases (*P* > 0.99) owing to matching, but not in terms of severity.

**Table 1:** General characteristics of participants

Characteristics	Herpes zoster (*n*, %)	Reference (*n*, %)	*P*-value*
Age (years)			1.000
20–24	1818 (3.1)	7272 (3.1)	
25–29	2790 (4.7)	11,160 (4.7)	
30–34	3409 (5.7)	13,636 (5.7)	
35–39	3889 (6.5)	15,556 (6.5)	
40–44	4623 (7.8)	18,492 (7.8)	
45–49	6189 (10.4)	24,756 (10.4)	
50–54	7964 (13.4)	31,856 (13.4)	
55–59	7239 (12.2)	28,956 (12.2)	
60–64	6273 (10.5)	25,092 (10.5)	
65–69	5783 (9.7)	23,132 (9.7)	
70–74	4611 (7.8)	18,444 (7.8)	
75–79	2798 (4.7)	11,192 (4.7)	
80–84	1381 (2.3)	5524 (2.3)	
85+	728 (1.2)	2912 (1.2)	
Sex			1.000
Male	23,677 (39.8)	94,708 (39.8)	
Female	35,818 (60.2)	143,272 (60.2)	
Income			1.000
1 (lowest)	9048 (15.2)	36,192 (15.2)	
2	8306 (14.0)	33,224 (14.0)	
3	9840 (16.5)	39,360 (16.5)	
4	13,072 (22.0)	52,288 (22.0)	
5 (highest)	19,229 (32.3)	76,916 (32.3)	
Region of residence			1.000
Urban	28,342 (47.6)	113,368 (47.6)	
Rural	31,153 (52.4)	124,612 (52.4)	
CCI score^†^			< 0.001*
0	21,182 (35.6)	100,129 (42.1)	
1	5942 (10.0)	24,823 (10.4)	
2	8187 (13.8)	30,511 (12.8)	
3	7804 (13.1)	27,512 (11.6)	
≥ 4	16,380 (27.5)	55,005 (23.1)	
Lymphoid neoplasm	90 (0.15)	212 (0.08)	< 0.001*

*Chi-square test; a *P*-value < 0.05 indicates significance

†CCI score was calculated without considering malignancies, including leukemia/lymphoma and metastatic solid tumors

*CCI* Charlson comorbidity index

The unadjusted and adjusted HRs of herpes zoster for lymphoid neoplasms were 1.68 (95% CI = 1.31–2.15) and 1.58 (95% CI = 1.23–2.02), respectively (*P* < 0.001 for both; Table 2). These findings was consistent with the unadjusted and adjusted HRs of herpes zoster for lymphoid neoplasms calculated 6 months after the index dates, as shown in an additional table [Additional file 1].

**Table 2:** Unadjusted and adjusted hazard ratios of herpes zoster for lymphoid neoplasms

Characteristics	Hazard ratios for lymphoid neoplasm			
	Unadjusted †	*P*-value*	Adjusted†‡	*P*-value*
Herpes zoster	1.68 (1.31–2.15)	< 0.001	1.58 (1.23–2.02)	< 0.001
Reference	1.00		1.00	

*Cox proportional hazards regression model; a *P*-value < 0.05 indicates significance

†Stratified model for age, sex, income, and region of residence

‡Model adjusted for the CCI score

*CCI* Charlson comorbidity index, *CI* confidence interval

On subgroup analyses according to age and sex, herpes zoster was associated with subsequent neoplasms in all subgroups (*P* < 0.05 for all, Table 3). The adjusted HRs were 1.53 (95% CI = 1.05–2.24) for participants < 60 years old, 1.58 (95% CI = 1.14–2.20) for participants ≥60 years old, 1.64 (95% CI = 1.16–2.31) for men, and 1.51 (95% CI = 1.06–2.16) for women.

**Table 3:** Hazard ratios of herpes zoster for lymphoid neoplasms according to age and sex

Characteristics	Hazard ratios for lymphoid neoplasm			
	Unadjusted †	*P*-value*	Adjusted†‡	*P*-value*
Age < 60 years (*n* = 189,605)				
Herpes zoster	1.71 (1.18–2.49)	0.005	1.53 (1.05–2.24)	0.026
Reference	1.00		1.00	
Age ≥ 60 years (*n* = 107,870)				
Herpes zoster	1.65 (1.19–2.29)	0.003	1.58 (1.14–2.20)	0.006
Reference	1.00		1.00	
Men (*n* = 118,385)				
Herpes zoster	1.75 (1.24–2.47)	0.001	1.64 (1.16–2.31)	0.005
Reference	1.00		1.00	
Women (*n* = 179,090)				
Herpes zoster	1.60 (1.20–2.28)	0.009	1.51 (1.06–2.16)	0.022
Reference	1.00		1.00	

* Cox proportional hazards regression model; a P-value < 0.05 indicates significance

† Stratified model for age, sex, income, and region of residence

‡ Adjusted model for the CCI score

*CCI* Charlson comorbidity index, *CI* confidence interval

On subgroup analysis according to the subtypes of lymphoid neoplasms, herpes zoster was associated with an increased risk of lymphoid neoplasms such as Hodgkin’s disease, diffuse NHL, and multiple myeloma/malignant plasma cell neoplasms according to the unadjusted analysis (*P* < 0.05 for all, Table 4). However, the adjusted HRs of herpes zoster were statistically significant only for Hodgkin’s disease and multiple myeloma/malignant plasma cell neoplasms (P < 0.05 for both). These adjusted HRs were 3.23 (95% CI = 1.17–8.93) for Hodgkin’s disease and 2.17 (95% CI = 1.33–3.54) for multiple myeloma/malignant plasma cell neoplasms.

**Table 4:** Hazard ratios of herpes zoster for lymphoid neoplasms according to the latters’ subtypes

Characteristics	Hazard ratio for lymphoid neoplasm			
	Unadjusted†	*P*-value*	Adjusted†‡	*P*-value*
Hodgkin’s disease (ICD-10 code = C81)				
Herpes zoster	3.34 (1.21–9.23)	0.020	3.23 (1.17–8.93)	0.024
Reference	1.00		1.00	
Follicular (nodular) non-Hodgkin’s lymphoma (ICD-10 code = C82)				
Herpes zoster	0.99 (0.28–3.52)	0.990	0.96 (0.27–3.41)	0.948
Reference	1.00		1.00	
Diffuse non-Hodgkin’s lymphoma (ICD-10 code = C83)				
Herpes zoster	1.61 (1.06–2.45)	0.025	1.51 (0.99–2.29)	0.055
Reference	1.00		1.00	
Peripheral and cutaneous T cell lymphomas (ICD-10 code = C84)				
Herpes zoster	1.49 (0.58–3.82)	0.402	1.42 (0.55–3.63)	0.467
Reference	1.00		1.00	
Other and unspecified types of non-Hodgkin’s lymphoma (ICD-10 code = C85)				
Herpes zoster	1.38 (0.93–2.06)	0.108	1.30 (0.88–1.94)	0.192
Reference	1.00		1.00	
Malignant immunoproliferative diseases (ICD-10 code = C88)				
Herpes zoster	1.66 (0.58–4.70)	0.343	1.51 (0.53–4.31)	0.441
Reference	1.00		1.00	
Multiple myeloma and malignant plasma cell neoplasm (ICD-10 code = C90)				
Herpes zoster	2.39 (1.47–3.89)	< 0.001	2.17 (1.33–3.54)	0.002
Reference	1.00		1.00	
Lymphoid leukemia (ICD-10 code = C91)				
Herpes zoster	1.02 (0.34–3.05)	0.975	1.01 (0.34–3.02)	0.988
Reference	1.00		1.00	

* Cox proportional hazards regression model; a P-value < 0.05 indicates significance

† Stratified model for age, sex, income, and region of residence

‡ Adjusted model for the CCI score

*CCI* Charlson comorbidity index, *CI* confidence interval, *ICD-10* International Statistical Classification of Diseases and Related Health Problems, tenth edition

In the analysis of the full cohort (i.e., without matching), the rate of subsequent lymphoid neoplasms was not significantly different in the 2 groups: 0.15% (92/59,926) in the herpes zoster group and 0.14% (980/689,524) in the reference group (*P* = 0.479) [see the table in Additional file 2]. The adjusted HR of herpes zoster for lymphoid neoplasms was 1.65 (95% CI = 1.32–2.06, *P* < 0.001) [as shown in the table in Additional file 3).

The nested case control study that investigated the history of herpes zoster showed that the lymphoid neoplasm group had a higher rate of patients with a previous history of herpes zoster (7.4% [92/1249]) than did the reference (lymphoid neoplasm-free) group (5.1% [254/4996], *P* < 0.05) [data are shown in a table in Additional file 4]. The adjusted OR of lymphoid neoplasms for previous herpes zoster was 1.45 (95% CI = 1.12–1.87, P < 0.05) [see the table in Additional file 5]. On subgroup analyses according to age or sex, lymphoid neoplasm affliction was significantly associated with a previous history of herpes zoster in participants ≥60 years old and in men, with adjusted ORs of 1.38 (95% CI = 1.01–1.87) and 1.49 (95% CI = 1.04–2.12) with P < 0.05 for both [shown in the table in Additional file 6]. All other subgroups tended to show such as association as well.

## Discussion

Our study showed that herpes zoster was significantly associated with an increased risk of subsequent lymphoid neoplasms (0.15% vs. 0.08% in the reference group) among the adult Korean population. This association was also confirmed in 3 additional analyses with 6-month wash-out periods, a full cohort analysis with no matching for reference participants, and a nested case-control study to investigate previous histories of herpes zoster in participants with and without lymphoid neoplasms. The associations persisted when analyzing subgroups categorized by age and sex. Herpes zoster was associated with an increased risk of Hodgkin’s disease and of multiple myeloma/malignant plasma cell neoplasms.

The overall risk of lymphoid neoplasms after herpes zoster infection was lower than that found in studies of Western populations, which also demonstrated an association between herpes zoster and subsequent lymphoid (or hematologic) neoplasms [1, 7]; however, the risk was on par with that recently reported by 2 Taiwanese groups [2, 9]. Previous studies by Western groups have shown rather inconsistent data; Ragozzini et al. and Fueyo et al. found that herpes zoster did not increase the risk of cancer or could not serve as a marker for occult neoplasms [11, 25]. In contrast, Sørensen et al. showed that the relative risks of hematological cancers including NHL, multiple myeloma, and leukemia were higher in patients who were previously hospitalized for herpes zoster (3.8 [95% CI = 1.9–6.7], 4.8 [95% CI = 21.9–9.4], and 2.8 [95% CI = 1.3–5.1], respectively), whereas the relative risk for all cancer types was only 1.3 (95% CI = 1.1–1.5) while that for solid cancers was not statistically significant [7]. The difference in the relative risks between our study and that of Sørensen et al. might have resulted from differences in methodologies (as mentioned above) and/or ethnicities. While their study was also based on a national registry, they included only patients who were hospitalized for herpes zoster, whereas we included both hospitalized patients and those who visited outpatient clinics. As there have been no published sets of factors to which relative risks should be adjusted, we were not able to compare our results to Sørensen et al.’s directly. Their study period (1977–1996) long predated ours, which may also have influenced the results. Cotton et al. also showed that the HR for hematological neoplasms in patients with herpes zoster was 3.02 (95% CI = 2.52–4.06), even though they did not consider lymphoid and myeloid neoplasms separately [1]. The maximum follow-up period in their study was 5 years whereas ours was 12 years; this may also have contributed to the difference in results.

Two recent studies using the national database in Taiwan suggested an increased risk of lymphoid neoplasms subsequent to herpes zoster in Asian populations; the reported HR was lower than that reported by the aforementioned Western groups but similar to ours, supporting both an association between herpes zoster and subsequent lymphoid neoplasms as well as an ethnicity-related factor. Lui et al. reported an HR of 1.68 (95% CI = 1.35–2.42) for lymphoid neoplasms [2], while Wang et al. reported HRs of 1.28 (95% CI = 0.83–1.89) and 1.31 (95% CI = 0.03–7.31) for NHL and Hodgkin’s disease, respectively, although without statistical significance [9]. Both studies were based on a very large number of populations extracted from the national health insurance program database. Lui et al. did not perform subgroup analyses according to age, sex, or lymphoid neoplasm subtypes; therefore, we cannot directly compare our results to theirs. However, Wang et al. did perform such subgroup analyses and showed that the risk of multiple myeloma increased only in patients with herpes zoster who were 60–79 years old. These contrasting data might be because of differences in ethnicity (Han vs. Korean) or the method of study subject inclusion (they included subjects who were hospitalized or visited outpatient clinics once or more, whereas our subjects were required to have done the same at least twice).

The increased risk of lymphoid neoplasms in subjects with herpes zoster may be attributed to decreased immunological surveillance that causes both herpes zoster and cancer simultaneously [26, 27]. However, because some cancers, including lymphomas, can be clinically undetectable for a relatively long time while herpes zoster symptoms can manifest sooner [8], the detection of herpes zoster may simply precede the detection of cancers chronologically. Another explanation could be asymptomatic occult cancers that actually arise first and cause immune system impairment that ultimately leads to herpes zoster [8], even though the herpes zoster manifests earlier. A third explanation could be that the zoster virus directly causes malignant transformation through biological mechanisms [28]. Infectious agents may induce chronic inflammation, forming reactive oxygen and nitrogen species that ultimately result in DNA damage that leads to carcinogenesis [29]. They also transform host cells directly by inserting oncogenes into the host genome, inhibiting tumor suppressors, or promoting mitosis [28]. Lastly, infectious agents may suppress immunity, resulting in decreased immune surveillance for cancers [30]. Nevertheless, whether herpes zoster itself increases the risk of lymphoid neoplasms or is merely an indicator of the immune impairment that precedes lymphoid neoplasms could not be determined in our study; further studies are required to establish the biological relationship between these 2 diseases.

Our data also suggest that the association between herpes zoster and subsequent lymphoid neoplasms is not as strong among Asian populations as it is in Western counterparts, which is consistent with 2 previous studies conducted in Taiwan. The genetic susceptibility of people from different ethnicities may explain these observations. Currently, almost all residents in South Korea are of a single ethnic background; likewise, Lui et al. and Wang et al. indicated that most Taiwanese are Han Chinese [2, 9]. Another less likely but possible explanation could be differences in the diagnostic modalities used in different countries.

The strengths of our study include that (i) it was a representative, large-scale sample from a national cohort database, (ii) it comprised well-defined study populations using stringent inclusion criteria, and (iii) concurrent multiple approaches were used to demonstrate the association between herpes zoster and subsequent lymphoid neoplasm development. The database consists of 1 million subjects with a maximum follow-up period of 12 years. We were able to investigate more participants than the number required per our power calculation because of the size of the database, which made our study the largest such study of its kind to date. As the dataset is based on claims made to the HIRA, the nationwide compulsory health insurance system, this approach minimizes the risk of recall bias or missing data [31]. Another strength of this study was our inclusion of well-defined populations; we included participants who were treated for herpes zoster ≥2 times or who were treated with antiviral medication ≥1 time(s) to treat herpes zoster. Patients with herpes zoster received their diagnoses in either an inpatient or outpatient clinic, which reduced the chance of selection bias that would arise from including only the most severely affected patients. Lastly, the results of our longitudinal follow-up cohort study using a matching process to select reference participants were supported by our other approaches, including another cohort study with matched reference participants that included a 6 month wash-out period, a full cohort study without matching, and a nested case-control study. Therefore, our finding of an association between herpes zoster infection and the subsequent diagnosis of lymphoid neoplasms in the Korean population is reliable.

One limitation of our study is that we could not adjust the data for some factors that may have affected the prevalence of lymphoid neoplasms, including obesity, smoking, alcohol use, and dietary habits. Nevertheless, the roles of such factors in lymphomagenesis are not considered as important as they are in other solid tumors; therefore, our results would very likely not differ appreciably had we taken such factors into account. Another limitation is that we did not adjust the data for any previous exposures to other types of infectious agents that are known to trigger lymphoid neoplasms, such as Epstein-Barr virus and human T cell leukemia/lymphotropic virus type 1. However, since the incidences of these viruses (especially the latter) in the adult population are very low, incorporating such data would likely not have affected our results. Nevertheless, additional studies that take into account laboratory data of other possible causative infectious agents may provide deeper insights regarding this matter.

## Conclusion

Our study demonstrated that herpes zoster is associated with an increased risk of subsequent lymphoid neoplasms irrespective of age and sex in the adult Korean population. Specifically, Hodgkin’s disease and plasma cell neoplasms are significantly associated with herpes zoster.

## Supplementary information

**Additional file 1: Table S1.** Unadjusted and adjusted hazard ratios (95% confidence intervals) of herpes zoster for lymphoid neoplasms 6 months after the index dates.

**Additional file 2: Table S2.** General characteristics of all participants.

**Additional file 3: Table S3.** Unadjusted and adjusted hazard ratios (95% confidence intervals) of herpes zoster for lymphoid neoplasms in all participants.

**Additional file 4: Table S4.** General characteristics of participants.

**Additional file 5: Table S5.** Unadjusted and adjusted odds ratios (95% confidence intervals) of lymphoid neoplasm development in patients with a previous history of herpes zoster.

**Additional file 6: Table S6.** Unadjusted and adjusted odds ratios (95% confidence interval) of lymphoid neoplasms for subjects with a previous history of herpes zoster.

## Data Availability

The data used for this study are available from the Korea National Health Insurance Sharing Service (https://nhiss.nhis.or.kr), subject to their requirements and fees.

## Abbreviations

CCI: Charlson comorbidity index
CI: Confidence interval
HIRA: Health Insurance Review and Assessment Service
HR: Hazard ratio
ICD-10: International Statistical Classification of Diseases and Related Health Problems, tenth edition
OR: Odds ratio
