# Supplementary material for: Increased risk of lymphoid malignancy in patients with herpes zoster: a longitudinal follow-up study using a national cohort
Source: BMC Cancer. 2019 Nov 27;19:1148. doi: 10.1186/s12885-019-6349-y (PMC6882027; doi:10.1186/s12885-019-6349-y)
Supplement: Supplementary file 1 — Additional file 1: Table S1. Unadjusted and adjusted hazard ratios (95% confidence intervals) of herpes zoster for lymphoid neoplasms 6 months after the index dates. [file 12885_2019_6349_MOESM1_ESM.docx]

**Additional file 1: Table S1.** Unadjusted and adjusted hazard ratios (95% confidence interval) of herpes zoster for lymphoid neoplasms 6 months after the index dates

| Characteristics | | Hazard ratios for lymphoid neoplasms | | | |
| --- | --- | --- | --- | --- | --- |
|  |  | Unadjusted† | P-value* | Adjusted†‡ | P-value* |
| Herpes zoster | | 1.79 (1.37–2.32) | <0.001 | 1.67 (1.29–2.18) | <0.001 |
| Reference | | 1.00 |  | 1.00 |  |

* Cox proportional hazards regression model; a P-value <0.05 indicates significance.

† Stratified model for age, sex, income, and region of residence.

‡ Adjusted model for the Charlson comorbidity index score.
