# Supplementary material for: Increased risk of lymphoid malignancy in patients with herpes zoster: a longitudinal follow-up study using a national cohort
Source: BMC Cancer. 2019 Nov 27;19:1148. doi: 10.1186/s12885-019-6349-y (PMC6882027; doi:10.1186/s12885-019-6349-y)
Supplement: Supplementary file 2 — Additional file 2: Table S2. General characteristics of all participants. [file 12885_2019_6349_MOESM2_ESM.docx]

**Additional file 2: Table S2.** General characteristics of all participants

| Characteristics | | | | Herpes zoster (n, %) | | Control (n, %) | | P-value | |
| --- | --- | --- | --- | --- | --- | --- | --- | --- | --- |
| Age (years old) | | | |  | |  | | <0.001* | |
|  | | 20-24 | | 1,831 (2.9) | | 81,654 (7.7) | |  | |
|  | | 25-29 | | 2,805 (4.4) | | 82,620 (7.8) | |  | |
|  | | 30-34 | | 3,421 (5.4) | | 93,979 (8.9) | |  | |
|  | | 35-39 | | 3,895 (6.1) | | 84,582 (8.0) | |  | |
|  | | 40-44 | | 4,627 (7.2) | | 87,369 (8.2) | |  | |
|  | | 45-49 | | 6,193 (9.7) | | 65,895 (6.2) | |  | |
|  | | 50-54 | | 7,970 (12.5) | | 47,259 (4.5) | |  | |
|  | | 55-59 | | 7,248 (11.3) | | 37,546 (3.5) | |  | |
|  | | 60-64 | | 6,294 (9.8) | | 37,254 (3.5) | |  | |
|  | | 65-69 | | 5,828 (9.1) | | 28,137 (2.7) | |  | |
|  | | 70-74 | | 4,695 (7.3) | | 19,158 (1.8) | |  | |
|  | | 75-79 | | 2,878 (4.5) | | 12,453 (1.2) | |  | |
|  | | 80-84 | | 1,448 (2.3) | | 7,225 (0.7) | |  | |
|  | | 85+ | | 793 (1.2) | | 4,393 (0.4) | |  | |
| Sex | | | |  | |  | | <0.001* | |
|  | | Male | | 23,828 (39.8) | | 344,186 (49.9) | |  | |
|  | | Female | | 36,098 (60.2) | | 345,338 (50.1) | |  | |
| Income | | | |  | |  | | <0.001* | |
|  | | 1 (lowest) | | 9,145 (15.3) | | 115,549 (16.8) | |  | |
|  | 2 | | 8,385 (14.0) | | 113,178 (16.4) | |  | |  |
|  | 3 | | 9,913 (16.5) | | 134,529 (19.5) | |  | |  |
|  | 4 | | 13,142 (21.9) | | 154,352 (22.4) | |  | |  |
|  | 5 (highest) | | 19,341 (32.3) | | 171,916 (24.9) | |  | |  |
| Region of residence | | |  | |  | | 0.120 | |  |
|  | Urban | | 28,592 (47.7) | | 331,271 (48.0) | |  | |  |
|  | Rural | | 31,334 (52.3) | | 358,253 (52.0) | |  | |  |
| CCI score^†^ | | |  | |  | | <0.001* | |  |
|  | 0 | | 21,260 (35.5) | | 338,792 (49.1) | |  | |  |
|  | 1 | | 5,959 (9.9) | | 88,329 (12.8) | |  | |  |
|  | 2 | | 8,220 (13.7) | | 92,493 (13.4) | |  | |  |
|  | 3 | | 7,870 (13.1) | | 68,460 (9.9) | |  | |  |
|  | ≥ 4 | | 16,617 (27.7) | | 101,450 (14.7) | |  | |  |
| Lymphoid neoplasm | | | 92 (0.15) | | 980 (0.14) | | 0.479 | |  |

*Chi-square test; a P-value <0.05 indicates significance.

†The CCI score was calculated without considering malignancies such as leukemias/lymphomas and metastatic solid tumors.

CCI, Charlson comorbidity index.
