# Supplementary material for: Increased risk of lymphoid malignancy in patients with herpes zoster: a longitudinal follow-up study using a national cohort
Source: BMC Cancer. 2019 Nov 27;19:1148. doi: 10.1186/s12885-019-6349-y (PMC6882027; doi:10.1186/s12885-019-6349-y)
Supplement: Supplementary file 3 — Additional file 3: Table S3. Unadjusted and adjusted hazard ratios (95% confidence intervals) of herpes zoster for lymphoid neoplasms in all participants. [file 12885_2019_6349_MOESM3_ESM.docx]

**Additional file 3: Table S3.** Unadjusted and adjusted hazard ratios (95% confidence interval) of herpes zoster for lymphoid neoplasms in all participants

| Characteristics | | Hazard ratios for lymphoid neoplasms | | | |
| --- | --- | --- | --- | --- | --- |
|  |  | Unadjusted† | P-value* | Adjusted†‡ | P-value* |
| Herpes zoster | | 2.79 (2.24-3.47) | <0.001 | 1.65 (1.32-2.06) | <0.001 |
| Reference | | 1.00 |  | 1.00 |  |

* Cox-proportional hazard regression model; a P-value <0.05 indicates significance.

†Adjusted model for age, sex, income and region of residence.

‡CCI score calculated without considering malignancies such as leukemias/lymphomas and metastatic solid tumors.

CCI, Charlson comorbidity index.
