# Supplementary material for: Increased risk of lymphoid malignancy in patients with herpes zoster: a longitudinal follow-up study using a national cohort
Source: BMC Cancer. 2019 Nov 27;19:1148. doi: 10.1186/s12885-019-6349-y (PMC6882027; doi:10.1186/s12885-019-6349-y)
Supplement: Supplementary file 4 — Additional file 4: Table S4. General characteristics of participants. [file 12885_2019_6349_MOESM4_ESM.docx]

**Additional file 4: Table S4.** General characteristics of participants

| Characteristics | | | | Lymphoid neoplasms (n, %) | | Reference (n, %) | | P-value* | |
| --- | --- | --- | --- | --- | --- | --- | --- | --- | --- |
| Age (years) | | | |  | |  | | 1.000 | |
|  | | 20–24 | | 32 (2.6) | | 128 (2.6) | |  | |
|  | | 25–29 | | 35 (2.8) | | 140 (2.8) | |  | |
|  | | 30–34 | | 49 (3.9) | | 196 (3.9) | |  | |
|  | | 35–39 | | 64 (5.1) | | 256 (5.1) | |  | |
|  | | 40–44 | | 76 (6.1) | | 304 (6.1) | |  | |
|  | | 45–49 | | 115 (9.2) | | 460 (9.2) | |  | |
|  | | 50–54 | | 138 (11.1) | | 552 (11.1) | |  | |
|  | | 55–59 | | 121 (9.7) | | 484 (9.7) | |  | |
|  | | 60–64 | | 140 (11.2) | | 560 (11.2) | |  | |
|  | | 65–69 | | 154 (12.3) | | 616 (12.3) | |  | |
|  | | 70–74 | | 152 (12.2) | | 608 (12.2) | |  | |
|  | | 75–79 | | 89 (7.1) | | 356 (7.1) | |  | |
|  | | 80–84 | | 62 (5.0) | | 248 (5.0) | |  | |
|  | | 85+ | | 22 (1.8) | | 88 (1.8) | |  | |
| Sex | | | |  | |  | | 1.000 | |
|  | Male | | 726 (58.1) | | 2,904 (58.1) | |  | |  |
|  | Female | | 523 (41.9) | | 2,092 (41.9) | |  | |  |
| Income | | |  | |  | | 1.000 | |  |
|  | 1 (lowest) | | 223 (17.9) | | 892 (17.9) | |  | |  |
|  | 2 | | 162 (13.0) | | 648 (13.0) | |  | |  |
|  | 3 | | 197 (15.8) | | 788 (15.8) | |  | |  |
|  | 4 | | 256 (20.5) | | 1,024 (20.5) | |  | |  |
|  | 5 (highest) | | 411 (32.9) | | 1,644 (32.9) | |  | |  |
| Region of residence | | |  | |  | | 1.000 | |  |
|  | Urban | | 610 (48.8) | | 2,440 (48.8) | |  | |  |
|  | Rural | | 639 (51.2) | | 2,556 (51.2) | |  | |  |
| CCI score^†^ | | |  | |  | | <0.001 | |  |
|  | 0 | | 117 (9.4) | | 1,923 (38.5) | |  | |  |
|  | 1 | | 198 (15.9) | | 472 (9.5) | |  | |  |
|  | 2 | | 248 (19.9) | | 640 (12.8) | |  | |  |
|  | 3 | | 240 (19.2) | | 597 (12.0) | |  | |  |
|  | ≥4 | | 446 (35.7) | | 1,364 (27.3) | |  | |  |
| Herpes zoster | | | 92 (7.4) | | 254 (5.1) | | 0.002 | |  |

*Chi-square test; a P-value <0.05 indicates significance.

†The CCI score was calculated without considering any malignancies, including leukemias/lymphomas and metastatic solid tumors.

CCI, Charlson comorbidity index.
