# Supplementary material for: Increased risk of lymphoid malignancy in patients with herpes zoster: a longitudinal follow-up study using a national cohort
Source: BMC Cancer. 2019 Nov 27;19:1148. doi: 10.1186/s12885-019-6349-y (PMC6882027; doi:10.1186/s12885-019-6349-y)
Supplement: Supplementary file 5 — Additional file 5: Table S5. Unadjusted and adjusted odds ratios (95% confidence intervals) of lymphoid neoplasm development in patients with a previous history of herpes zoster. [file 12885_2019_6349_MOESM5_ESM.docx]

**Additional file 5: Table S5.** Unadjusted and adjusted odds ratios (95% confidence intervals) of lymphoid neoplasm development in patients with a previous history of herpes zoster.

| Characteristics | | Odds ratios for herpes zoster | | | |
| --- | --- | --- | --- | --- | --- |
|  |  | Unadjusted† | P-value* | Adjusted†‡ | P-value* |
| Lymphoid neoplasm | | 1.50 (1.17-1.93) | 0.002 | 1.45 (1.12-1.87) | 0.004 |
| Reference | | 1.00 |  | 1.00 |  |

*Conditional logistic regression analysis; a P-value <0.05 indicates significance.

†Stratified for age, sex, income, and region of residence.

‡Model is adjusted for the Charlson comorbidity index score.
