# Supplementary material for: Increased risk of lymphoid malignancy in patients with herpes zoster: a longitudinal follow-up study using a national cohort
Source: BMC Cancer. 2019 Nov 27;19:1148. doi: 10.1186/s12885-019-6349-y (PMC6882027; doi:10.1186/s12885-019-6349-y)
Supplement: Supplementary file 6 — Additional file 6: Table S6. Unadjusted and adjusted odds ratios (95% confidence interval) of lymphoid neoplasms for subjects with a previous history of herpes zoster. [file 12885_2019_6349_MOESM6_ESM.docx]

**Additional file 6: Table S6.** Unadjusted and adjusted odds ratios (95% confidence interval) of lymphoid neoplasms for subjects with a previous history of herpes zoster.

| Characteristics | | Odds ratios for herpes zoster | | | |
| --- | --- | --- | --- | --- | --- |
|  |  | Unadjusted† | P-value* | Adjusted†‡ | P-value* |
| Age <60 years (n = 3,150) | | | | | |
|  | Lymphoid neoplasms | 1.78 (1.14–2.77) | 0.011 | 1.55 (0.98–2.44) | 0.060 |
|  | Reference | 1.00 |  | 1.00 |  |
| Age ≥60 years (n = 3,095) | | | | | |
|  | Lymphoid neoplasms | 1.39 (1.03–1.88)* | 0.034 | 1.38 (1.01–1.87) | 0.041 |
|  | Reference | 1.00 |  | 1.00 |  |
| Men (n = 3,630) | | | | | |
|  | Lymphoid neoplasms | 1.62 (1.14–2.29) | 0.007 | 1.49 (1.04–2.12) | 0.029 |
|  | Reference | 1.00 |  | 1.00 |  |
| Women (n = 2,615) | | | | | |
|  | Lymphoid neoplasms | 1.39 (0.97–1.99) | 0.076 | 1.39 (0.97–2.00) | 0.077 |
|  | Reference | 1.00 |  | 1.00 |  |

* Conditional logistic regression analysis; a P-value <0.05 indicates significance.

†Stratified for age, sex, income, and region of residence.

‡Model is adjusted for the Charlson comorbidity index score.
